# The Use of Elevenies as a Novel Tool in Organic Chemistry Teaching for Pharmacy Students

**DOI:** 10.3390/pharmacy14030069

**Published:** 2026-05-08

**Authors:** Daniel Baecker

**Affiliations:** Department of Pharmaceutical and Medicinal Chemistry, Institute of Pharmacy, Freie Universität Berlin, Königin-Luise-Straße 2+4, 14195 Berlin, Germany; d.baecker@fu-berlin.de

**Keywords:** drug, education, elevenie, interdisciplinary, learning aid, organic chemistry, pharmacy, poem, student motivation, teaching method

## Abstract

Teaching organic chemistry is also important for pharmacists to understand the synthesis and mechanism of action of organic drug molecules. Unfortunately, organic chemistry is considered one of the most difficult subjects. This impression affects students’ motivation. To provide students with a learning aid and hopefully boost their motivation, this pilot study tested the integration of 28 elevenies—a special form of short poem—during a semester in an organic chemistry lecture for pharmacists. An anonymous and voluntary questionnaire was conducted at the end of the lecture sessions to assess perceptions of the use of elevenies as a teaching tool. Overall, the student feedback on the implementation of elevenies was positive. In general, students felt (with nearly 94% agreement) that a wider variety of learning methods, such as elevenies, should be incorporated into university teaching. They found elevenies, a type of literature, suitable for summarizing content of organic chemistry, a natural science. The majority (about 65%) stated that they secretly looked forward to the presentation of the elevenies during the lecture, indicating an increase in motivation. In addition, 83% of the respondents wanted to adduce elevenies to repeat and learn the teaching material. However, only about 20% could imagine writing elevenies themselves as part of the learning process. With 94% approval, the respondents gave a clear vote to retain elevenies in future semesters. This suggests the students’ perception of elevenies as an educational tool. Their ease of use could certainly be extended to other subject areas, provided that the topics addressed are not too complex.

## 1. Introduction

Among pupils at school and students at university, chemistry is considered one of the most difficult subjects to learn [1]. There are various reasons for this [2], including teaching practices and students’ lack of motivation [3]. The latter challenges lecturers, especially when chemistry is taught as a minor rather than a major subject. This is the case, for example, with studying pharmacy, as it covers many chemistry-related subjects [4]. In chemistry, the area of organic chemistry is said to be the most difficult [5,6]. A study by Eticha and Ochonogor [7] revealed that organic synthesis reactions and the mechanisms behind them were the most demanding for students.

These overall circumstances served as the motivation for the research presented within this manuscript. Therefore, the intention was to use a simple tool in a lecture to boost both students’ motivation and their feasibility to learn organic chemistry content. This can generally be achieved, for example, through the use of new digital technologies such as podcasts or interactive videos [8,9,10]. However, these might be hampered by the laborious effort of their production and potential technical issues for both teachers and students.

**Figure 1:**
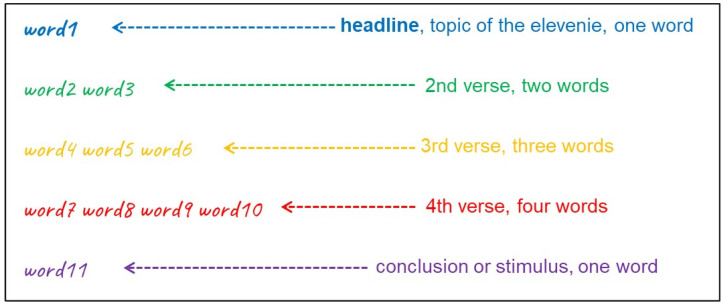
Schematic depiction of the defined structure of an elevenie, in total consisting of five verses comprising eleven words.

Poems are a technically accessible method for summarizing and condensing educational content, fostering academic progression [11]. In particular, elevenies are short poems with a fixed form consisting of a total of eleven words (see Figure 1). The first line encompasses one single word and indicates the topic of the elevenie. The following lines comprise two, three, and then four words, respectively. The last line consists of just one word again and often serves as a stimulus for the conclusion.

In the current study, elevenies were integrated into an organic chemistry lecture. The aim of this pilot study was to investigate how pharmacy students evaluate the use of elevenies as an educational tool to facilitate organic chemistry teaching.

## 2. Materials and Methods

### 2.1. Curricular Setting

The pilot study was conducted at the Institute of Pharmacy, Freie Universität Berlin, Germany during the summer term 2024. In particular, the integration of elevenies was investigated as part of the course “Chemistry and Medicinal Chemistry for Pharmacists”. According to the curriculum, this course is intended for pharmacy students of the second semester (of eight semesters in total). It represents a lecture, which aligns to the course “Chemistry including the Analysis of organic drugs, excipients, and contaminants”. The latter is a laboratory course in which the pharmacy students practically perform organic reactions in the laboratory, relevant for drug synthesis. This course comprises the actual exam, while the theoretical concepts of the synthesis reactions and basics of organic chemistry are taught in the course “Chemistry and Medicinal Chemistry for Pharmacists” and are therefore covered in the exam. According to the curriculum, successfully passing this exam is mandatory for the pharmacy students to proceed to the next semester (their third semester).

### 2.2. Study Implementation

Before conducting the pilot study, including the voluntary and anonymous survey, this project was reported to the assistant to the associate dean for academic affairs. Permission was granted. No further ethical approval for this non-interventional study was deemed necessary by the quality management division of the university since students were informed in advance, since the questionnaire did not collect any personal data, and because the results cannot be used to draw conclusions about individual students.

During the summer term 2024, a total of 92 students attended the lecture “Chemistry and Medicinal Chemistry for Pharmacists”. Before using the first elevenie in the lecture, the purpose was introduced to the students. The intention of the pilot study on university teaching methods was explained, i.e., hopefully providing a source that helps with learning organic chemistry content. This idea originated as an action on the student feedback from the previous semester. They mentioned difficulties in memorizing basic organic chemistry concepts and mainly in distinguishing different name reactions, as well as the mechanisms behind them. There was a brief explanation of what elevenies are in general, that they will be integrated bit by bit in the lecture, and that there will be a voluntary and anonymous questionnaire at the end of the semester. The intention to publish the results of the survey was mentioned when introducing the study to the students.

The survey asked the students about their perceptions regarding the implementation of elevenies in the organic chemistry lecture. The questionnaire was bilingual (German and English) and consisted of eleven statements.

The questions were created by the lecturer himself without further verification or validation by any other party except for the author. No tools of generative artificial intelligence were used to generate the questions for the survey. Students were asked to indicate their responses by checking boxes on a 5-point Likert scale, to establish if they strongly agree (score 1), agree (score 2), disagree (score 4), or strongly disagree (score 5) with the statements. It was also possible to express a neutral (score 3) attitude towards the statements. The survey took place by handing a printed questionnaire to the students during one of the last sessions. A break between the session was used for the purpose of filling out the questionnaire. The lecturer was not present during this break. There was neither an incentive nor a disadvantage for students when participating in the survey or not doing so, respectively.

The analysis of the survey was descriptive and included a qualitative count of the respective levels of (dis)agreement with the statements and the calculation of the mean Likert score. 

### 2.3. Elevenies

During the lecture “Chemistry and Medicinal Chemistry for Pharmacists”, a total of 28 elevenies in German, written by the lecturer himself, were used. No tools of generative artificial intelligence were used to poetize the elevenies. Some examples of elevenies, translated into English for the purpose of this publication, can be found in Appendix A. In the summer semester 2024, the lecture was held over 31 sessions, meaning that, on average, nearly one poem was presented per lecture session. The elevenies were mainly given at the beginning of a lecture. Because of their condensed form, they were used to repeat important content from the previous session. In general, the spaced use of repetition tools was found beneficial with respect to the increase in pharmacy students’ performance [12]. The presentation of the elevenies was often accompanied by a picture or a diagram of a general chemical equation.

The content of the elevenies covered general topics in organic chemistry (e.g., hyperconjugation, Hückel’s rule, electrophilic aromatic substitution [S_E_Ar], arines, carboxylic acids, ketals, polymerization) or specific name reactions (e.g., Diels–Alder, Grignard, Knoevenagel, Kolbe–Schmitt, Mannich, Michael, Vilsmeier–Haack). In the latter case, the name (though often used as a double name) was treated as a single word, so to speak, to preserve the formality of the elevenies. Furthermore, the elevenies based on name reactions often ended with the international non-proprietary name of a pharmaceutical drug. For this purpose, compounds whose synthesis route includes the name reaction described in the elevenie were used.

This approach was specifically designed to establish a concrete connection to pharmacy in terms of referring to particular drug molecules. This gives the course a certain legitimacy, since pharmaceutical drugs must, after all, be synthesized using organic chemistry.

## 3. Results

A total of 66 students participated in the survey. Based on the number of students enrolled in the course (i.e., 92 students), this corresponds to 72%. Table 1 presents the detailed results of the survey.

The first question was aimed at the students’ previous experience with elevenies, both passively through reading and actively through writing. The “strongly disagree” and “disagree” responses indicated that almost 70% of the students had not previously encountered elevenies. Only about 20% agreed and strongly agreed with that statement.

Based on the results of the first question, the findings of the second item seemed obvious. Specifically, the question asked whether respondents were already familiar with the use of elevenies as a creative learning tool. Only 4.5% strongly agreed and 13.6% agreed, while 22.7% disagreed and another 45.4% strongly disagreed in terms of familiarity with elevenies as a learning tool.

This means that just over two-thirds (about 68%) of students are not familiar with the use of elevenies as an element in the context of teaching. In contrast, the response to the third statement is particularly interesting. In that case, students were asked whether they agreed or disagreed that alternative methods (e.g., elevenies) should be used more frequently in university education. A mean score of all responses of exactly 1.50 on the 5-point Likert scale indicates a value that falls, so to speak, exactly between “agree” (34.8%) and “strongly agree” (59.1%). This means that almost 94% of the students surveyed are indirectly expressing a preference for more innovative learning methods.

This raises the question of whether elevenies, in particular, are suitable as a representative example of such tools. Item 4 addressed whether elevenies are appropriate for summarizing learning content. About 32% agreed with this statement, and even 52% of the students strongly agreed, while 15% gave a neutral response.

In the fifth statement, students assessed whether elevenies—which, given their genre, constitute a form of literature and would typically be classified rather as a subject of literary studies—are also suitable for a subfield of natural science (i.e., organic chemistry). Half of the students strongly agreed that the use of elevenies in the natural science of organic chemistry is beneficial. Another 39% agreed, and 9% were neutral. Only one of the 66 respondents strongly disagreed with the statement.

Interest in the elevenies can also be attributed to curiosity about their presentation in the lectures. The use typically occurred at the beginning of a lecture session. In the context of reviewing and summarizing key content from previous lecture sessions, elevenies were often utilized. Most students, about one-third (34.8%), strongly agreed with the statement that they were already somewhat excited about new elevenies. The response “agree” was given by 30.3%, while a quarter (25.8%) had a neutral attitude toward this. With a mean score of 2.15 and the anticipation of new elevenies among approximately 65% of the students, this tool appears to have a motivating effect.

In addition to increasing motivation for organic chemistry-related content, the aim of this pilot study was to use the elevenies as a tool to help students repeat and learn the teaching material. Just over half of the respondents (53.0%) strongly agreed that elevenies would be used for this purpose. An additional 30.3% agreed. This translated into a low mean score on the 5-point Likert scale, with a value of 1.62, indicating that students were willing to accept the tool as a learning aid.

Given the formally prescribed limit of eleven words, elevenies are highly condensed. However, the contextual relationships in organic chemistry often seem too complex to be adequately described with so few words. Item 8 addressed the point that eleven words may not be sufficient to cover all the important aspects of name reactions. With a mean score of 3.05, a fairly balanced opinion between agreement and disagreement on this issue emerged. Around 30% of respondents each selected “agree” (29.5%), “neutral” (34.8%), and “disagree” (27.3%). One questionnaire included the note “depends”, which likely indicates that it depends on the particular name reaction whether eleven words are sufficient to describe it.

The survey revealed that students were somewhat looking forward to the elevenies (item 6) and were highly willing to make use of them during the learning phase (item 7). However, the length of only eleven words does not seem ideally suited for covering all the necessary content (item 8). This suggests that students might expand the elevenies beyond the eleven-word limit in terms of content. The question of whether students can imagine writing elevenies themselves in the future was actually raised in item 9. Most respondents (40.9%) selected “disagree”. Since 12.1% also strongly disagreed, nearly half of those surveyed are opposed to writing their own elevenies. A total of 27.3% hold a neutral standpoint, meaning that just under 20% would actually be willing to compose their own elevenies.

Item 10 asked whether students found it stimulating that several of the elevenies about name reactions ended with a specific drug name as a stimulus. On this point, the students’ responses were fairly neutral, with a mean Likert score of 2.58 and the majority selecting “neutral” (35.4%). Nevertheless, 27.7% agreed that they found this encouraging and a further 18.5% even strongly agreed. One student added the handwritten comment “did not know about it”.

The final item in the survey asked respondents to evaluate the claim that the use of elevenies should be continued in future semesters. This statement received the highest level of agreement (mean score of 1.33) among all the survey items. Nearly three-quarters (72.7%) strongly agreed, and an additional 21.3% agreed, together accounting for 94.0%. Strikingly, none of the participants expressed any degree of disagreement. This appears to confirm the success of integrating elevenies into an organic chemistry lecture as part of this pilot study.

## 4. Discussion

### 4.1. Interpretation

Even though the combination of chemistry and poetry may initially seem contradictory, given that they belong to the natural sciences and literary studies, respectively, there may be some lecturers who see this as a perfect fit. Precisely because some concepts in chemistry are very abstract, metaphorical descriptions are used to explain them, paving the way for poetry, which also often makes use of metaphorical language [13]. This kind of symbiosis has a long history. Rayner-Canham discussed examples from the period around the turn of the 18th and 19th centuries, in which female chemists in particular used poetry to describe, document, or even critique scientific content [14]. It was highlighted that poems can be applied not only in school, but also at university and even in one’s future professional life. In the current study (item 5), almost 90% of the respondents expressed a positive view that elevenies, a specific type of poem, can be used in a science discipline, i.e., organic chemistry. When compared with the mean score of 1.64 and the fact that no student selected disagree for this statement, it could be assumed that the single strongly dissenting student potentially has a fundamental lack of interest in any form of poetry. However, the overall result of this statement has to be interpreted with caution. It is unclear to which extent pharmacy students are able to evaluate the usefulness of literary methods in the natural sciences.

By bringing chemistry and poetry together in an interdisciplinary yet unconventional way, the gap between natural science and art can be bridged. This fosters a hedonistic attitude, because it makes chemistry more interesting and enjoyable, thereby boosting students’ motivation [15,16]. Furthermore, the synergy between chemistry and poetry can also help to stimulate students’ curiosity [17]. This is certainly evident in the current study (item 6) as well, in which about 65% expressed a positive attitude toward the statement that they were secretly looking forward to the presentation of new elevenies. This curiosity can ultimately further commitment and learning, as reported for another subject, i.e., conservation science, previously [18].

Overall, about 94% of the respondents indirectly stated that alternative methods such as elevenies should be used much more frequently in university teaching (item 3). The demand for the use of poetry more often in teaching chemistry content also emerged in the study by Araujo et al. [17]. This may be due to the fact that such educational methods have so far been employed only in a very limited way. In the specific case of elevenies, this hypothesis is confirmed in the present pilot study by the fact that approximately two-thirds of the students were not yet familiar with elevenies as a creative learning tool (item 2).

In general, the respondents have little experience with elevenies, either through reading or writing (item 1). The outcome of this item demonstrated that elevenies are somewhat novel to the students. It might be particularly the lack of experience in writing this type of poem that can act as a hindrance to becoming active as poets themselves. This is also reflected in the outcome of the survey (item 9), which shows that only about 20% can imagine writing elevenies themselves as part of the learning process. Just over half expressed a negative attitude toward this. This result is somewhat surprising, especially given that: (i) nearly 94% would be in favor of using novel tools such as elevenies more frequently (item 3); and (ii) about 83% of the students surveyed indicated that they would use the elevenies to repeat and learn the organic chemistry course content (item 7).

The learning process is actually intended to be enhanced through students’ independent writing of elevenies. By actively engaging with the scientific content in this way, students would deal with the subject matter more deeply [19]. In doing so, creativity and imagination would be stimulated and the students would interconnect ideas, recognize patterns, develop a critical perspective on matters, and build connections, thereby establishing links and improving learning [20]. Because students put their thoughts into a concrete form through writing, this also helps to train their communication skills [20]. Furthermore, writing poems can serve as a reflective means in the learning process, helping to gain a better understanding of students’ experiences by putting them into words [21].

This apparent reluctance among students to write elevenies themselves emerged solely from the survey conducted as part of the study. The active component of encouraging students to write elevenies was not an element of the current pilot study and thus represents a limitation or, more accurately, an approach for a future intervention. Similarly, the current study did not measure the effect on learning outcomes. However, the pilot study was designed to assess the overall perception. This was indeed demonstrated very effectively, as 94% of respondents expressed support for continuing to use this tool in the following semesters. The remaining 6% had a neutral attitude, and none of the respondents opposed keeping the elevenies (item 11).

Another constraint of using elevenies is that the eleven words of the poem might not be sufficient to cover all the essential aspects of a topic. This is reflected by the responses to item 8 of the survey. Elevenies seem difficult to apply to more complex relationships, such as the directing effects in an electrophilic aromatic substitution reaction. The same might apply to multifaceted relationships, for example in pharmacological contexts. In contrast, with a mean score of 1.67 in item 4, the students generally confirmed that elevenies are capable of condensing content.

For the organic chemistry lecture, students generally viewed elevenies as a positive development, particularly given the encouraging overall results that suggest it should be continued to use elevenies. This indicates that elevenies as an educational tool were accepted by the students. To the best of the author’s knowledge, however, there have been no reports in the scientific literature to date on the use of this particular type of poem in university education. They not only provide added value for students, but also serve as a smart and enriching tool for lecturers in their teaching practices. In any case, it is easy to implement. A few students certainly proved this by writing an elevenie in their final exam using their own initiative.

### 4.2. Limitations

In general, a positive perception by the students regarding the use of elevenies is indicated from the outcomes of the survey. However, the pilot study also has some limitations.

These refer, for example, to the questionnaire and the performance of the survey. In the current study, the items of the survey were not verified or validated by other experts except for the author himself. This might result in the data not being fully conclusive. After validation, it would be warranted that the content actually intended (i.e., validity) is measured during the survey and that the measurement is consistent and reproduceable (i.e., reliability) [22].

The validity of the content could be obtained through reviewing by a panel of experts [23]. The performance of a pilot testing, i.e., studying the questionnaire in a small cohort of students, provides information about the understandability of the items and thus allows for their subsequent refinement if necessary [24]. Internal consistency and reliability of a survey could be quantified using the so-called Cronbach’s alpha [25]. This coefficient is a statistical measure describing the capability of various items in a questionnaire to assess one particular feature. There are even further parameters that could be used to evaluate the quality of a survey [26].

Since the current study was designed as a pilot project, comprehensive validation was not carried out. However, given the absence of referring to such established survey design frameworks, the data obtained have to be interpreted with caution. This weakens the significance of the outcomes to a certain extent.

The questionnaire was filled out by the students in a printed form. This was performed during a break in one of the lecture sessions. Beforehand, the lecturer handed out the questionnaires to the students. This self-administered procedure might lead to a social desirability bias [27]. This bias describes the tendency of the respondents to vote in favor of the elevenies as their lecturer put a lot of effort in creating the poems and implementing them in the organic chemistry course. Compared with an in-person interview, the written survey provides a certain degree of physical distance to the interviewer, thus reducing this distortion [28]. However, to even better avoid this bias, an online survey conducted by the student outside the lecture hall would be recommendable [29]. Carrying out an anonymous survey also contributes to reducing the social desirability bias. However, this was already implemented in the current study.

Another limitation of the presented pilot study could be seen in the lack of data concerning the effect on the learning outcome. As stated above, measuring such an effect was not the intention of the pilot study. However, the meaningfulness of the research would be strengthened when data from a control group (i.e., no use of elevenies) or following a pre/post design would have been adduced [30]. This would allow for the deduction of an effect of elevenies as an educational tool on the learning outcome not only in a descriptive manner but also based on inferential statistics. The latter would allow for the drawing of stronger conclusions [31].

To summarize, given these limitations of the pilot study, it can be concluded that future, well-designed investigations are necessary. Such studies can help not only to indicate students’ perceptions of elevenies but also to confirm a potentially positive effect on the learning process.

## 5. Conclusions

In pharmacy studies, education in chemistry plays a significant role. Unfortunately, chemistry is generally considered one of the most challenging subjects, particularly the field of organic chemistry. However, knowledge in this subject is essential for pharmacists to understand the synthesis and modes of action of organic drug molecules. To boost the motivation of pharmacy students and provide them with a learning aid, a total of 28 elevenies covering basic knowledge and name reactions were integrated into a 31-session organic chemistry lecture for pharmacists as part of a pilot study. Finally, the students’ perceptions were surveyed.

Despite—or perhaps especially because of—a lack of exposure to these tools and an implied desire for more diverse learning methods, the tool was very well received. Although elevenies represent a form of literature, a large majority considered them useful in a natural science context, thereby bridging the gap between science and the arts. In their feedback, the respondents indicated that elevenies are a suitable tool for summarizing content and that they would use them in their repetition and learning processes. Since there was a certain sense of anticipation among the students regarding the presentation of the elevenies, they seem to increase their interest and, consequently, their motivation. However, the students expressed that they would hardly be willing to create elevenies themselves as part of the learning process. This is an area with great potential, as active encouragement to write would lead to a more reflective and thus deeper engagement with the subject matter.

Overall, the students voted clearly in favor of retaining elevenies in future semesters. This demonstrates that elevenies are an interesting learning tool. They not only offer advantages for students but also enrich the lecturer’s teaching practice through their simple implementation. An expansion to other subject areas beyond organic chemistry seems likely, provided that elevenies are not adduced to convey content that is too complex.

This publication is dedicated to Jürgen Braunschweig on the occasion of his 90th birthday.

## Figures and Tables

**Table 1:** Feedback on the survey regarding the integration of elevenies in an organic chemistry lecture, presented as absolute and relative (in %) responses of 66 participants (*n* = 66).

No.	Statement	Strongly Agree (Score 1)	Agree (Score 2)	Neutral (Score 3)	Disagree (Score 4)	Strongly Disagree (Score 5)	Mean Score
1	I have often read or even written elevenies myself before.	5 (7.6%)	9 (13.6%)	6 (9.1%)	20 (30.3%)	26 (39.4%)	3.80
2	I was already familiar with the use of elevenies as a creative learning tool.	3 (4.5%)	9 (13.6%)	9 (13.6%)	15 (22.7%)	30 (45.4%)	3.91
3	I am of the opinion that university teaching in general should use different methods, such as elevenies, more often.	39 (59.1%)	23 (34.8%)	2 (3.0%)	2 (3.0%)	0 (0%)	1.50
4	In principle, I consider elevenies to be a suitable means of summarizing teaching content.	34 (51.5%)	21 (31.8%)	10 (15.2%)	1 (1.5%)	0 (0%)	1.67
5	Although organic chemistry belongs to the natural sciences, I think the use of elevenies (a form of literature) is good.	33 (50.0%)	26 (39.4%)	6 (9.1%)	0 (0%)	1 (1.5%)	1.64
6	I was secretly looking forward to new elevenies at the start of a new lecture.	23 (34.8%)	20 (30.3%)	17 (25.8%)	2 (3.0%)	4 (6.0%)	2.15
7	I will use the elevenies to repeat or learn the teaching material.	35 (53.0%)	20 (30.3%)	8 (12.1%)	3 (4.5%)	0 (0%)	1.62
8	The eleven words of an elevenie are not enough to cover all the important aspects of a name reaction.	1.5 ^1^ (2.3%)	19.5 ^1^ (29.5%)	23 (34.8%)	18 (27.3%)	4 (6.0%)	3.05
9	I could imagine writing elevenies myself in the future while learning.	5 (7.6%)	8 (12.1%)	18 (27.3%)	27 (40.9%)	8 (12.1%)	3.38
10	I find it encouraging that a number of the elevenies about name reactions ended with the name of a specific drug. ^2^	12 (18.5%)	18 (27.7%)	23 (35.4%)	9 (13.8%)	3 (4.6%)	2.58
11	The use of elevenies should be continued in future semesters.	48 (72.7%)	14 (21.3%)	4 (6.0%)	0 (0%)	0 (0%)	1.33

^1^ One student put a cross between the two answer choices “strongly agree” and “agree” and made the handwritten note of “The middle of the two”. When evaluating this particular response, the handwritten addition was understood to indicate the mathematical mean (i.e., middle) between the two choices “strongly agree” and “agree”. With respect to the merely descriptive analysis of the survey (e.g., calculation of the mean Likert score), the response was therefore split equally between these two options, thus representing the mean. ^2^ One student did not answer this question, thus resulting in *n* = 65.

## Data Availability

The original contributions presented in this study are included in the article. Further inquiries can be directed to the corresponding author.

## Appendix A. Examples of Elevenies

Below are examples of some of the elevenies used in the pilot study, presented here in their English translations. The elevenies were written by Daniel Baecker (Institute of Pharmacy, Freie Universität) in German and translated into English for the purpose of this publication. Because the names of chemicals and vocabularies are spelled differently, the number of words after translation into English is not exactly the same as in German.

**Cannizzaro**
aldehyde disproportionation
strong bases present
carboxylic-acid and primary alcohol
hydride-shift

**Chichibabin**
from pyridine
formation of 2-aminopyridine
by heating with sodium-amide
S_N_Ar

**Diels–Alder**
concerted cycloaddition
four plus two
diene reacts with dienophile
Biperiden

**Hückel**
planar system
cyclic and conjugated
number π-electrons is (4n + 2)
aromatic

**Hyperconjugation**
electronic interaction
explanation of stabilization
complete and incomplete orbitals
delocalization

**Ketal**
geminal diether
formed from ketone
by addition of alcohols
Triamcinolone-hexacetonide

**Knoevenagel**
α,β-unsaturated acids
malonic-acid and catalyst
aldehyde or ketone reacts
Gabapentin

**Leuckart–Wallach**
reductive amination
reduction by formic-acid
“heating carbonyl with amine”
Methamphetamine

**Mannich**
methylene-activated carbonyl
aminomethylation with formaldehyde
maximum of secondary amine
Tramadol

**Michael**
methylene-activated compound
basic catalyst present
nucleophilic addition to alkene
Atracurium-besilate

**Ozonolysis**
1,3-dipolar addition
primary-ozonide and secondary-ozonide
decomposition under reducing conditions
Harries

**Polymerization**
from monomers
to repeating units
radical, cationic, or anionic
tacticity

**Vilsmeier–Haack**
for formylation
DMF and POCl_3_
chloromethylene iminium as electrophile
Ketorolac

**Wolff–Kishner**
strong base
heating with hydrazine
aldehyde or ketone reduced
alkane
